# The Association between Databases for Indexing Studies Intended for an Exercise Meta-Analysis of Arthritis Randomized Controlled Trials

**DOI:** 10.1155/2012/624830

**Published:** 2012-08-09

**Authors:** George A. Kelley, Kristi S. Kelley

**Affiliations:** Meta-Analytic Research Group, School of Medicine, Department of Community Medicine, Robert C. Byrd Health Sciences Center, West Virginia University, P.O. Box 9190, Morgantown, WV 26506-9190, USA

## Abstract

*Objective*. The purpose of this study was to determine the database indexing of randomized controlled trials (RCTs) for a meta-analysis addressing the effects of exercise on pain and physical function in adults with arthritis and other rheumatic diseases (AORD). *Methods*. The number, percentage, and 95% confidence intervals (CIs) for included articles at initial and follow-up periods were calculated from PubMed, EMBASE, CENTRAL, CINAHL, SPORTDiscus, and DAO databases. The number needed to review (NNR) was also calculated along with the number of articles retrieved by expert review. Cross-referencing from reviews and included articles also occurred. *Results.* Thirty-four of 36 articles (94.4%, 95% CI, 81.3–99.3) were located by database searching. PubMed and CENTRAL yielded 32 of 36 articles (88.9%, 73.9–96.9). Two articles not identified in any of the other databases were found in either CINAHL or SPORTDicsus. Two other articles were located by scanning the reference lists of review articles. The NNR ranged from 2 (CINAHL) to 118 (SPORTDiscus). More articles were identified in EMBASE at follow-up (36%, 12.1–42.2 versus 86.1%, 70.5–95.3). *Conclusions*. Searching multiple databases and cross-referencing from reviews was important for identifying RCTs addressing the effects of exercise on pain and physical function in adults with AORD.

## 1. Introduction

 Systematic reviews, with or without meta-analysis, are increasingly used to examine the effects of exercise in participants with arthritis and other rheumatic diseases (AORD). For example, a recent PubMed search up to May 25, 2012 and limited to systematic reviews and meta-analyses addressing the effects of exercise in participants with AORD yielded a total of 193 citations. One of the major steps when conducting a systematic review, with or without a meta-analysis, is a comprehensive search for all relevant articles on the topic of interest [1]. When conducting such a search, it is important to be thorough but efficient. Since the vast majority of articles included in systematic reviews are retrieved from electronic database searches [2, 3], knowledge regarding the indexing of articles from each database is important. Recent research that examined previous meta-analyses of randomized controlled trials (RCTs) of the orthopedic surgical literature reported that more than 97% of included articles were retrieved from three electronic databases: MEDLINE, EMBASE, and Cochrane [2]. However, such results are likely to vary depending on the topic of choice. To the best of our knowledge, no previous study has attempted to assess the indexing of included articles for an exercise and AORD meta-analysis. This is surprising given the large number of meta-analytic citations on this topic. The authors have recently compiled a database of articles for a meta-analysis of RCTs addressing the effects of exercise on pain and physical function in adults with arthritis and other rheumatic diseases (AORDs). The purpose of this study was to determine the percentage of included articles that were indexed in the various databases. 

## 2. Materials and Methods

 Articles included in the meta-analysis had to be (1) RCTs with an exercise-only group (aerobic, strength training, or both), (2) community-deliverable exercise interventions ≥4 weeks, (3) a comparative control group (non-intervention, usual care, attention control), (4) adults aged 18 years and older with either rheumatoid arthritis, osteoarthritis, fibromyalgia, lupus, gout, or ankylosing spondylitis, (5) studies published in peer-reviewed journals as well as master's theses and dissertations, (6) articles published in any language between January 1, 1980 and January 1, 2008, and (7) data available for pain and/or physical function. 

 Articles were retrieved by (1) searching six different electronic databases (PubMed, EMBASE, Cochrane Central Register of Controlled Clinical Trials (CENTRAL), Cumulative Index to Nursing and Allied Health Literature (CINAHL), SPORTDiscus and Dissertation Abstracts Online (DAO), (2) cross-referencing from included articles, (3) cross-referencing from review articles, and (4) expert review (Dr. Miriam Nelson, Tufts University, personal communication, June 13, 2008). We included DAO because we were interested in studies that may not have been published but were available in thesis or dissertation format. We excluded Google Scholar based on a search that resulted in 25,500 citations after excluding patents and including only biology and medicine in the search. The rationale for this exclusion was based on fact that screening such a large number of citations was beyond the resources available for this and most other projects. 

 While the keywords and combination of keywords varied depending upon the database being searched, terms commonly used included “exercise,” “arthritis,” “rheumatic,” “rheumatoid arthritis,” “osteoarthritis,” “fibromyalgia,” “lupus,” “gout,” and “ankylosing spondylitis.” A list of the user queries for each database is shown in (Supplement 1 available online at doi:10.1155/2012/624830.) With the exception of EMBASE, initial searches were conducted by the second author. For EMBASE, the initial search was conducted by an expert on exercise and arthritis who had access to such (Dr. Jennifer Hootman, personal communication, January 22, 2008).

 Studies were selected for inclusion by both authors, independent of each other. The authors then met and reviewed every selection. Discrepancies were resolved by consensus. If consensus could not be reached, two experts on exercise and AORD acted as arbitrators (Dr. Jennifer Hootman and Dr. Dina Jones, personal communication). Using Cohen's kappa statistic, the overall agreement rate prior to correcting discrepancies was 0.89. 

 For this small study, the number, percentage, and 95% confidence intervals for included articles derived from the initial search of each database was calculated. As a follow-up, the number, percentage, and 95% confidence intervals for articles actually indexed in each database was also calculated. This was accomplished by entering various combinations of each missing article's title, author name(s), journal name, and publication year into each database [4]. For the DAO database, only the one included dissertation was searched for [5]. With the exception of EMBASE, the identification of articles at follow-up was conducted by the first author. Follow-up searching in EMBASE was conducted by an expert on exercise and arthritis (Dr. Jennifer Hootman, personal communication, December 30, 2009). 

 In addition to calculating the number, percentage, and 95% confidence intervals for included articles from initial and follow-up searches, the number needed to review (NNR) was calculated for each database by dividing the total number of initial citations to be screened by the number of articles that were included. 

## 3. Results

**Figure 1:**
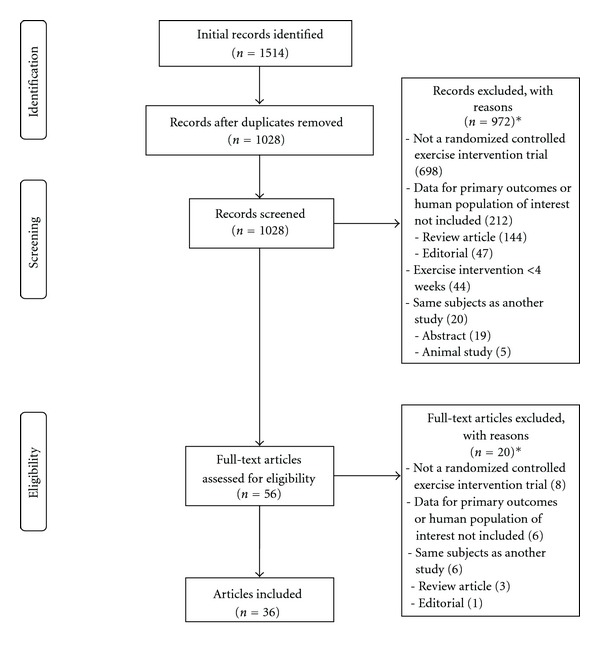
Flow diagram describing the search process. *, number of reasons exceeds the number of studies excluded because some studies excluded for more than one reason.

 A flow diagram describing the initial search process, including the number of studies examined, is shown in Figure 1. The results for initial and follow-up searches for included studies as well as the NNR are shown in Table 1, the reference list of included articles in Supplement 2 available online at doi:10.1155/2012/624830, and database indexing for each article from each database in Supplement 3 available online at doi:10.1155/2012/624830. 

### 3.1. Initial Search

 Thirty-four of 36 articles (94.4%, 95% CI, 81.3–99.3) that met the criteria for inclusion were located via initial database searches. Two articles (5.6%, 0.7–18.7) were located by scanning the reference lists of review articles [6, 7]. None were included based on scanning the reference lists of included articles or from expert review. All included articles were published in the English language. As can be seen in rows 1 through 3 of Table 1, the CENTRAL and PubMed databases yielded the greatest number of included articles followed by EMBASE, CINAHL, and SPORTDiscus. None were located using DAO. All studies initially identified in EMBASE were also identified in PubMed and/or CENTRAL. When combined, both the CENTRAL and PubMed databases yielded 32 of 36 articles (88.9%, 73.9–96.9). The crossover between the two databases was 75.0% (56.6–88.5). Excluding the two articles that were located by scanning the reference lists of reviews [6, 7], one article that was not available in PubMed, CENTRAL, or EMBASE was located using CINAHL [8] while another was identified using SPORTDiscus [9]. 

### 3.2. Follow-Up Searches

 When each database was searched for each article at follow-up (rows 4 through 6 of Table 1), the CENTRAL and PubMed databases yielded the greatest number of articles followed by EMBASE, CINAHL, and SPORTDiscus. With the exception of DAO, all follow-up searches resulted in the identification of a greater number of articles. This was particularly true for EMBASE (non-overlapping confidence intervals and 50.1% difference). For the two articles that were initially identified by scanning the reference lists of review articles but not found in any database [6, 7], one [6] was found in PubMed, EMBASE, CENTRAL, and CINAHL while the other [7] was located in PubMed, EMBASE, and CENTRAL. One article that was initially identified in SPORTDiscus was also found in PubMed, EMBASE, CENTRAL, and CINAHL at follow-up [9]. One other article continued to be limited in location to CINAHL [8]. 

### 3.3. Number Needed to Review (NNR)

 Row 7 of Table 1 includes data on the NNR. As shown, the largest NNR was from the SPORTDiscus database while the smallest was from CINAHL. 

## 4. Discussion

The results of this study suggest that searching multiple databases was important when trying to locate all RCTs addressing the effects of exercise on AORD in adult humans. This suggestion is based on the lack of 100% crossover between databases as well as the inability to locate, initially, articles that may reside in multiple databases but may only be identified by one. In addition, because our search strategies were unable to identify articles that were located in one or more databases but could not be identified by any of them suggests that scanning the reference lists of resources such as review articles was important. The inability to initially locate articles that existed in a database, especially EMBASE, was most likely the result of the queries developed and used to conduct the searches and/or the search filters provided by the database vendors. In the case of EMBASE, that large discrepancy in this study may have been the result of someone with less experience conducting the searches. 

 In contrast to database searching and scanning the reference lists of review articles, we did not find expert review or scanning the reference lists of included articles to be helpful. Given these findings, inclusion of these approaches may not be worthwhile. However, before any firm conclusions can be drawn, this should probably be tested using other systematic reviews. 

 Clearly, the two most important databases searched for the current study were CENTRAL and PubMed. While including EMBASE has been recommended [2], it did not yield any studies that could not be identified in CENTRAL and/or PubMed. However, since the focus of CENTRAL is on RCTs, it is unlikely to be very helpful for non-RCTs such as studies of test accuracy [3]. 

 With the exception of SPORTDiscus, the NNR appeared to be equitable for all other databases. Based on these findings, it might seem appropriate to suggest that SPORTDiscus not be searched given the large NNR and low yield. However, since the goal of a systematic review is to identify all articles that meet one's inclusion criteria and one article not initially identifiable by any other databases or approaches was located using SPORTDiscus [9], the inclusion of this database was important. In contrast, the inclusion of the DAO database was not important for this study. 

 Given that systematic reviews are increasingly used in the exercise and arthritis literature, thorough and efficient literature searches are important for conducting high-quality systematic reviews with or without meta-analysis. The results of the current study provide an important first step in that direction. 

 While the current study provides important information in relation to searching and retrieving exercise studies with respect to AORD, the generalization of the current findings to other systematic reviews on AORD may be limited. Additional research on optimal approaches in searching for relevant articles to include in systematic reviews on AORD is needed. For a much broader perspective on this important topic, one is referred to the recent work of others in relation to optimal search strategies for the identification and retrieval of studies for systematic reviews, with or without meta-analysis [10–17]. 

## 5. Conclusions

The results of this study suggest that searching multiple databases as well as cross-referencing from review articles was important for identifying RCTs addressing the effects of exercise on pain and physical function in adults with AORD. However, additional research in this area is needed before any firm recommendations can be made. 

## Figures and Tables

**Table 1:** Indexing of articles from database searches.

Variable	PubMed	EMBASE	CENTRAL	CINAHL	SPORTDiscus	DAO
Initial search						
* n* (%)	27 (75)	9 (36)	29 (80.6)	11 (30.6)	3 (8.3)	0 (0)
(95% CI)	(57.8–87.9)	(12.1–42.2)	(64.0–91.8)	(16.3–48.1)	(1.8–22.5)	(NA)
Follow-up search						
*n* (%)	34 (94.4)	31 (86.1)	35 (97.2)	19 (52.8)	6 (16.7)	0 (0)
(95% CI)	(81.3–99.3)	(70.5–95.3)	(85.5–99.9)	(35.5–69.6)	(6.4–32.8)	(NA)
NNR	9	12	16	2	118	NA

Notes: Total number of articles included = 36; CENTRAL: Cochrane Central Register of Controlled Clinical Trials; CINAHL: Cumulative Index to Nursing and Allied Health Literature; DAO: Dissertation Abstracts Online; *n* (%): number and percentage of articles; 95% CI: 95% confidence interval; NNR: Number needed to review, calculated by dividing the total number of citations to review per database divided by the number of studies included from the initial search; NA: not applicable.
